# Supplementary material for: Learning curve and surgical outcome of robotic assisted colorectal surgery with ERAS program
Source: Sci Rep. 2022 Nov 29;12:20566. doi: 10.1038/s41598-022-24665-w (PMC9709162; doi:10.1038/s41598-022-24665-w)
Supplement: Supplementary file 1 — Supplementary Information 1. [file 41598_2022_24665_MOESM1_ESM.zip › LSC Raw data-Ñ[▒K/089/P_ñw╝╨░O▒Kñσ.pdf]

[redacted]

Pathologic diagnosis:
1. Rectum, laparoscopic low anterior resection --- a. Adenocarcinoma, moderately to poorly differentiated, arising in a tubular adenoma. Tumor invaded to muscularis propria, and metastasized to perirectal lymph nodes (7/20). The bilateral cut ends are free of tumor. b. Hyperplastic polyp and diverticulum.
2. IMA root, dissection ---Unremarkable fibrovasculoadipose tissue.
3. Colon, cut end, distal, resection --- Chronic inflammation.

Ancillary study for diagnosis:
IHC stains for PMS2 and MSH6 are done on section G. All above markers are preserved.

Prognostic and predictive factor:
1. Tumor size: 3x2.8 cm.
2. Depth of invasion: Muscularis propria.
3. Histologic grade: G2: Moderately differentiated to G3: Poorly differentiated.
4. Angiolymphatic invasion: Not identified.
5. Perineural invasion: Not identified.
6. Tumor deposits (discontinuous extramural extension): Present, specify number of deposits: 1.
7. Macroscopic tumor perforation: Not identified.
8. Surgical Margins: a) Proximal Margin: Uninvolved by invasive carcinoma, distance of tumor from margin: 13 cm. b) Distal Margin: Uninvolved by invasive carcinoma, distance of tumor from margin: 1 cm. c) Radial or Mesenteric Margin: Uninvolved by invasive carcinoma, distance of tumor from margin:1.5 cm.
9. Lymph node status: Involved (7/20).
10. Extranodal involvement: Present.
11. Tumor regression grading S/P CCRT: No known presurgical therapy.
12. Type of polyp in which invasive carcinoma arose: Tubular adenoma.
13. Additional pathologic findings: Hyperplastic polyp and diverticulum.
14. Pathological TNM Stage: pT2N2b (According to the eighth edition, American Joint Committee on Cancer Staging Guidelines for Tumors).
15. TNM descriptors: Not applicable.

Gross description:
The specimen consists of 1) a segment of rectum, 17 cm in length, 2 cm and 3.2 cm in both cut end circumference, and perirectal soft tissue. A gray tan firm annular elevated and centrally ulcerative tumor, 3x2.8 cm, is noted, 1 cm to the nearest cut end. On cross section, the tumor invades to the muscular layer. The both cut ends are free of tumor grossly. 2) IMA root, a piece of tan yellow soft tissue, 3x3x0.5 cm. 3) distal cut end, a piece of tan mucosa-covered soft tissue, 1.5x1x0.5 cm. All for section: A) specimen 2 B) specimen 3; Representative part for section: C-K) specimen 1 [C) distal cut end D) proximal cut end E-G) tumor H-K) lymph nodes.

Note:
The interpretation of MMR panel and recommended studies are as below: (1) No loss of nuclear expression of MMR proteins: low probability of microsatellite instability-high (MSI-H) . (2) Please note: There are exceptions to the above IHC interpretations. These results should not be considered in isolation, and clinical correlation with genetic counseling is recommended to assess the need for germline testing.
#T-59600_2 #M-81403_2 3 1105 M0000F
#T-C4560_2 #M-81406_2 3 1105 000000

已電子簽章

請病患妥為保管，若不慎遺失需自負個人資料外洩責任！
